# Identifying early key influencing factors of positive results in the early screening for postpartum depression with interpretable machine learning

**DOI:** 10.3389/fpubh.2026.1761983

**Published:** 2026-05-15

**Authors:** Shusen Lin, Yan Wang, Xuefei Han, Xiaojing Wang, Yanxia Zhang, Wanyu Xu, Meiyu Chen, Xiaobing Xu, Huawei Li

**Affiliations:** 1 School of Nursing, Qingdao University, Qingdao, Shandong, China; 2 Qilu Hospital, Shandong University (Qingdao), Qingdao, China

**Keywords:** early influencing factors, machine learning, postpartum depression, recursive feature elimination, short-term longitudinal study

## Abstract

**Objective:**

Based on social ecosystem theory, this study explores the early key factors associated with postpartum depressive symptoms from multiple dimensions, and analyzes them using interpretable machine learning algorithms.

**Methods:**

This was a short-term longitudinal study. Using the convenience sampling method, pregnant women who were hospitalized in the obstetrics departments of two tertiary grade-A hospitals in Shandong Province, China, from July 2023 to January 2025 and met the inclusion and exclusion criteria were selected as the research subjects. Based on social ecosystem theory, candidate factors were discussed in expert meetings, and 53 candidate factors were finalized for the survey. After feature selection using recursive feature elimination combined with 10-fold cross-validation, early risk factors for EPDS screening-positive postpartum depressive symptoms were identified using logistic regression. The model construction was carried out using Python 3.12 to build 9 machine learning algorithm models and conduct internal and external validations. After selecting the best model based on various evaluation indicators, SHAP (Shapley Additive Explanations) analysis was conducted within the best model to obtain the global interpretability analysis.

**Results:**

After recursive feature elimination and logistic regression, six early risk factors for EPDS screening-positive postpartum depressive symptoms were identified: whether there was work pressure during maternity leave, whether forced eating occurred due to breastfeeding, the relationship between the mother and her husband, whether weight gain during pregnancy caused distress, the current sleep situation of the mother, and the degree of social support. After comparing the internal and external validation indicators of the 9 machine learning algorithm models, the LightGBM model was found to be the best model. Finally, the SHAP analysis was presented in the form of swarm plots and Polar Plots, which demonstrated the global feature importance ranking within the best model, the influence direction and distribution of each early risk factors, as well as the interaction pattern between feature values and their impacts.

**Conclusion:**

This study combines the social ecosystem theory and interpretable machine learning algorithms to explore the early risk factors for EPDS screening-positive postpartum depressive symptoms, which can provide a reference basis for the subsequent formulation of early risk management plans.

## Introduction

The concept of postpartum depression (PPD) was first proposed by Roland M in 1950 (1). It is a syndrome of mental disorders characterized by a core symptom cluster of emotional depression, loss of interest and pleasure, and reduced energy (2). It is the most common psychological complication after childbirth and a research hotspot both at home and abroad. It often occurs within 2 weeks after delivery, with symptoms typically most prominent at 4–6 weeks postpartum (3). Studies have shown that 80% of women will experience a brief postpartum depressive mood, and 15% of women will develop severe postpartum depression (4). Additionally, the global prevalence of postpartum depression is 14%, but in developing countries, especially in China, the prevalence rate is significantly higher than that in developed countries, reaching 21.4% (5).

PPD often fails to be timely and accurately identified and intervened after the mother is discharged from the hospital (6, 7), so early and precise identification and intervention during the hospital stay after delivery are particularly important. In existing studies, the early influencing factors of postpartum depression mainly involve three aspects: society, family, and the mother herself (8). However, due to differences in sample characteristics, research designs, and data analysis methods, the correlation weights and intensities of specific influencing factors vary (9, 10). Therefore, previous studies have lacked a method that can precisely identify and comprehensively analyze the early influencing factors of postpartum depression. Nowadays, the application of machine learning in postpartum depression is gradually maturing. Some scholars have used it to construct postpartum depression prediction models (11) to predict postpartum depression in mothers, and related studies have explored key influencing factors of maternal mental health through interpretable machine learning methods (12) to achieve early intervention. However, research on the early precise identification of postpartum depression is still scarce, and there is a lack of relevant theoretical support. Therefore, this study aims to comprehensively analyze the early risk factors for EPDS screening-positive postpartum depressive symptoms from four dimensions: individual, family, community and hospital, and society based on the theory of social ecology. This theory can help explore the influence of social ecological environment on individuals and among individuals. It can describe and analyze the influencing factors of individuals from multiple levels and perspectives. We applied machine learning methods to analyze the key influencing factors and conduct a global interpretability analysis of the influencing factors, providing a reliable reference for the early precise identification and intervention of postpartum depression.

## Methods

### Study design and participants

This was a short-term longitudinal study that employed convenience sampling. The training set included pregnant women who met the inclusion and exclusion criteria and were hospitalized in the obstetric departments of two tertiary grade-A general hospitals in Qingdao from July 2023 to October 2024. The test set was for temporal and spatial validation and also used convenience sampling. It included pregnant women who met the inclusion and exclusion criteria and were hospitalized in the obstetric departments of two tertiary grade-A general hospitals in Qingdao from November 2024 to January 2025. The training set was used for model development and internal validation, while the test set was used for external validation.

This study received ethical approval from the Ethics Committee of Qingdao Medical College, Qingdao University (QDU-HEC-2021114). Written informed consent was obtained from all participants prior to participation. Eligible participants were postpartum women who had experienced uncomplicated deliveries, were willing to take part in the study, and were able to complete the investigation. Women were excluded if they had a prior history of psychiatric disorders (e.g., depression), cognitive or intellectual impairments, or if their families had undergone major traumatic events or significant life changes between early pregnancy (first 6 months) and delivery.

The sample size of the training set was estimated based on the Kendall sample size estimation method (13), with the inclusion sample size being 5 to 10 times the number of independent variables. In this study, there were 53 candidate independent variables. Considering a 10% invalid questionnaire recovery rate, the minimum sample size was calculated to be at least 292 pregnant women. The internal validation was randomly split in an 8:2 ratio, with 80% for model building and 20% for internal validation. The training set ultimately included 563 valid research subjects. The test set was used for external validation, and its sample size is generally 1/4 to 1/2 of the modeling sample size (14–16). Considering a 10% invalid questionnaire recovery rate, the external validation group required at least 57 pregnant women. The test set ultimately included 236 valid research subjects.

### Instruments

#### The early risk factors for PPD questionnaire

This questionnaire is based on literature review and social-ecological theory, and analyzes the early influencing factors of postpartum depression from the individual level, family and interpersonal level, community and hospital level, and social level. After expert meetings for discussion and revision (inviting 2 obstetric medical experts and 2 obstetric clinical nursing experts, all 4 experts have over 15 years of working experience in this field and hold senior professional titles or above), 53 candidate influencing factors were obtained, which are as follows:

① Individual factors (micro): Maternal age, personality traits, educational level, economic income, and nutritional status (weight gain, whether folic acid is supplemented, etc.), whether there are complications or other diseases during pregnancy, whether it is a planned pregnancy, whether there are breastfeeding obstacles, maternal mental genetic factors, sleep status of pregnant and postpartum women, etc.

② Family and interpersonal factors (meso): Husband's age, educational level, economic income, the level and stability of the family's economic income, and the interpersonal relationships of the pregnant and postpartum women, whether there are people to confide in, whether the care and support system of the pregnant and postpartum women is guaranteed, whether there is sufficient care and support in terms of diet and daily life, whether the pregnant and postpartum women have experienced mental stimulating events, whether they have suffered from domestic violence, etc., family relationships of the pregnant and postpartum women, especially the support from their spouses; the evaluation of social support by the pregnant and postpartum women, the views of the family and the pregnant and postpartum women on the gender of the fetus and whether there is prejudice, the health status of the newborn, whether there are diseases, etc., the temperament type of the newborn (whether they are prone to crying or fussing).

③ Community and hospital factors (macro): The living conditions of the pregnant and postpartum women and whether it is convenient to seek medical treatment, whether the relationship between the family and the community neighbors is harmonious, the evaluation of the medical level of the designated maternity hospital, etc.

④ Social factors (macro): Work pressure of the pregnant and postpartum women, whether they have medical insurance, etc.

#### The Social Support Rating Scale (SSRS)

The Social Support Rating Scale was developed by Xiao Shuiyuan. It consists of 10 items and is divided into three dimensions: subjective support, objective support, and the utilization of support (17). The score range of this scale is from 13 to 62. A score above 45 indicates a high level of social support, 33 to 45 indicates a moderate level of social support, and 13 to 33 indicates a low level of social support. The reliability coefficient of this scale is 0.920. In this study, it was used to measure the degree of social support of the mothers in the above-mentioned questionnaire, and it was also used to measure along with it.

#### The Edinburgh Postnatal Depression Scale (EPDS)

The Edinburgh Postnatal Depression Scale (EPDS), initially developed by Cox et al. (18) in 1987, is used to assess postpartum depression, with a reliability coefficient of 0.87. This scale consists of 10 items that evaluate emotional and psychological feelings, such as mood, enjoyment, self-blame, anxiety, and insomnia. Each item is scored on a 4-point scale (0 to 3) based on the severity of the symptoms, with a total score range of 0 to 30. In this study, an EPDS score ≥10 was used as the cutoff for a positive screen for postpartum depression.

This cutoff has demonstrated acceptable sensitivity and specificity in multiple validation studies conducted among Chinese postpartum populations (11, 32). However, variation in EPDS cutoff values across studies may influence prevalence estimates; therefore, the screening-positive rate reported in this study should be interpreted in the context of the selected screening threshold.

### Data collection

Before the investigation, standardized instructions were provided to the participants to inform them of the purpose, significance and content of the study. After obtaining their informed consent, data collection was conducted through questionnaires and electronic medical record inquiries. Data from the participants were collected at two time points: from after delivery until discharge and 42 days postpartum. Considering that some information in the survey was sensitive, the researchers compiled the items and option scales of the above scales or questionnaires into electronic questionnaires and sent the links to the research subjects or invited them to scan the questionnaire QR codes to fill them out themselves. Some research subjects used paper questionnaires. The Early Risk Factors for PPD Questionnaire was filled out from after delivery until discharge, and the Edinburgh Postnatal Depression Scale was filled out by the researchers through a phone follow-up with the participants 4 to 6 weeks postpartum.

### Statistical analysis

Descriptive analysis of the training set and test set data was conducted using SPSS 29.0 software. All continuous variables were normally distributed and were presented as mean ± standard deviation (SD), while the categorical variables were presented as frequencies and percentages.

### Feature selection

Since each question in the questionnaire of this study was accompanied by a “must-answer” prompt, there are no missing values in the data of this study. Before feature selection, one-hot encoding was applied to unordered multi-class variables. Subsequently, recursive feature elimination (RFE) combined with 10-fold cross-validation was used for feature selection in the machine learning algorithm, and then logistic regression was used to identify key influencing factors.

As a greedy algorithm, RFE iteratively builds a base model to evaluate the importance of each feature, then ranks the features based on their importance scores and gradually eliminates the less relevant features (19). Unlike simple feature selection methods based on statistics or models, recursive feature elimination with cross-validation (RFECV) dynamically adjusts the feature subset through iterative elimination and cross-validation, effectively reducing the number of features while maintaining model accuracy.

### Model development and evaluation

Model construction was carried out using the Scikit-learn library in Python 3.12. Nine machine learning algorithm models suitable for classification outcomes were selected: Logistic Regression (LR), Decision Tree (CART), Random Forest (RF), K-Nearest Neighbor (KNN), eXtreme Gradient Boosting (XGBoost), Adaptive Boosting (AdaBoost), Gaussian Naive Bayes (GaussianNB), Light Gradient Boosting Machine (LightGBM), and Support Vector Machine (SVM). The training set data were randomly split into a modeling group and an internal validation group at a ratio of 8:2 using the train_test_split function. The models were adjusted through grid search for the best parameters, and finally, the models were tested using internal and external validation data, and various indicators were calculated, such as accuracy, precision, recall, F1 score, AUC value, and 95% CI. The accuracy of the model's predicted probabilities was evaluated using the calibration curve, and the clinical application value and net benefit of the model were evaluated using the decision curve analysis (DCA).

### Model explanation

SHAP (SHapley Additive exPlanations) is a model-agnostic interpretability method grounded in cooperative game theory, specifically derived from the Shapley value framework. It explains individual model predictions by decomposing the output into the additive contributions of each input feature relative to a baseline (expected) prediction. By calculating the marginal contribution of a feature across all possible feature coalitions, SHAP provides a theoretically consistent and locally accurate attribution of feature effects. This approach enables the interpretation of complex “black-box” machine learning models-such as ensemble tree methods or gradient boosting algorithms-within a unified additive explanation framework, ensuring both global and local interpretability.

In the present predictive modeling study, SHAP was applied to quantify feature importance, elucidate the directionality of predictor effects, and explore potential non-linear relationships between predictors and the outcome. Global SHAP summary analyses allowed ranking of variables according to their overall contribution to model output, while dependence patterns illustrated how variations in specific predictors influenced risk probability. Furthermore, individual-level SHAP explanations enabled decomposition of predicted risk into patient-specific driving factors, thereby enhancing clinical interpretability and supporting targeted risk stratification. Collectively, the integration of SHAP strengthened the transparency, clinical relevance, and translational utility of the developed prediction model.

## Results

### Descriptive analysis of maternal data

**Figure 1:**
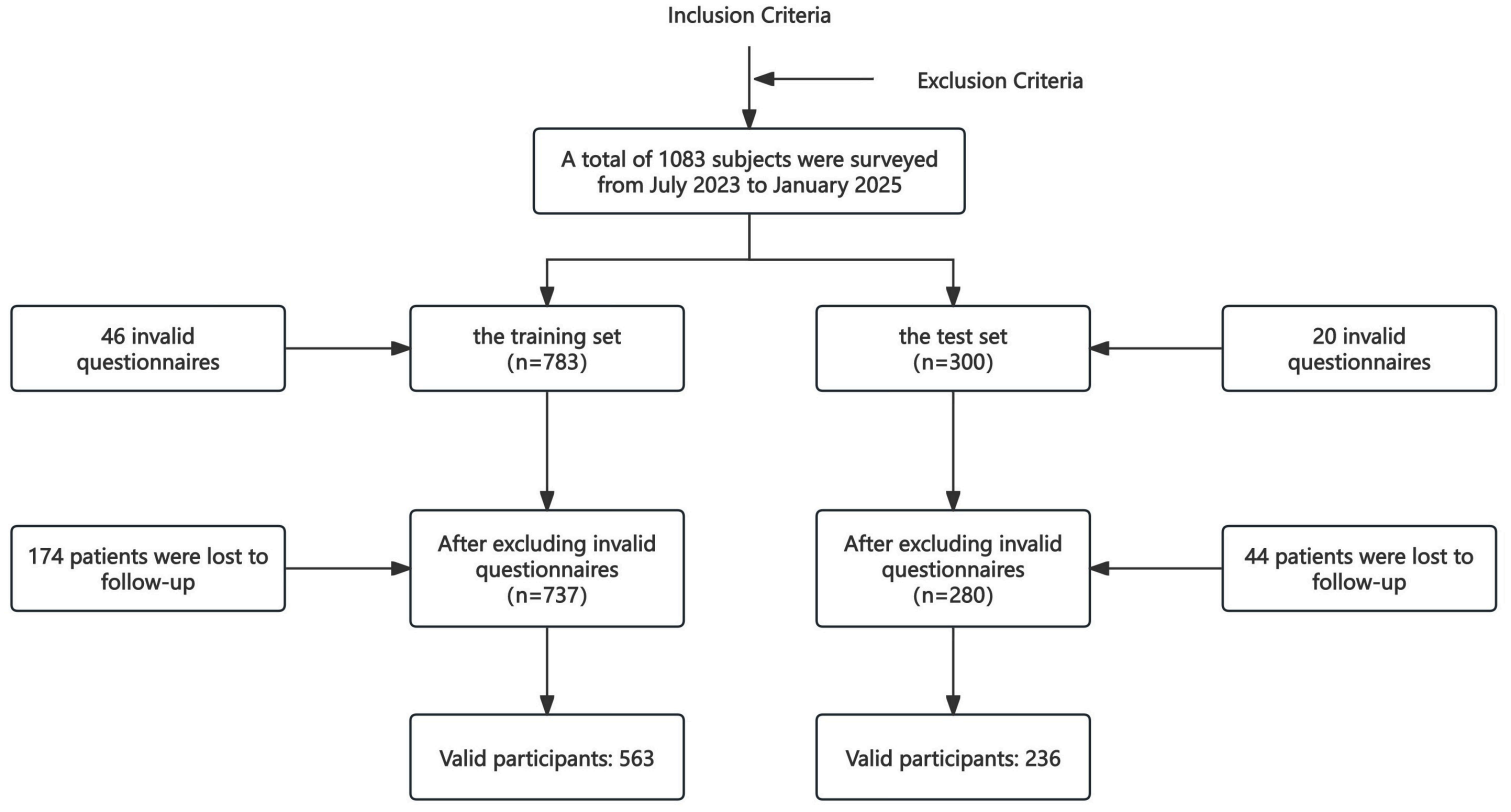
Data collection process for participants.

This study investigated a total of 1,083 postpartum women from July 2023 to January 2025, and ultimately obtained 799 valid questionnaire data. Among them, the training set included 563 postpartum women, with an EPDS screening-positive rate of 35.7%, and the test set included 236 postpartum women, with an EPDS screening-positive rate of 30%. The EPDS screening-positive rate (EPDS ≥ 10) among all 799 postpartum women was 34% (see Appendix for detailed descriptive statistics), and Figure 1 shows the flowchart of participant data collection.

### Screening of early influencing factor characteristics of PPD

**Figure 2:**
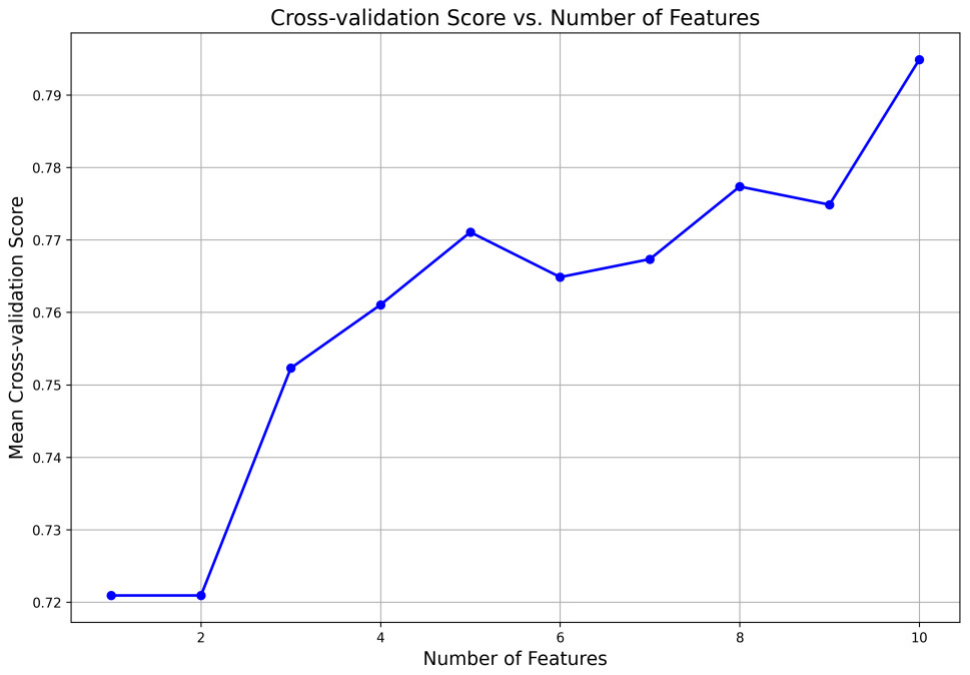
Optimal number of features selected by RFECV.

**Figure 3:**
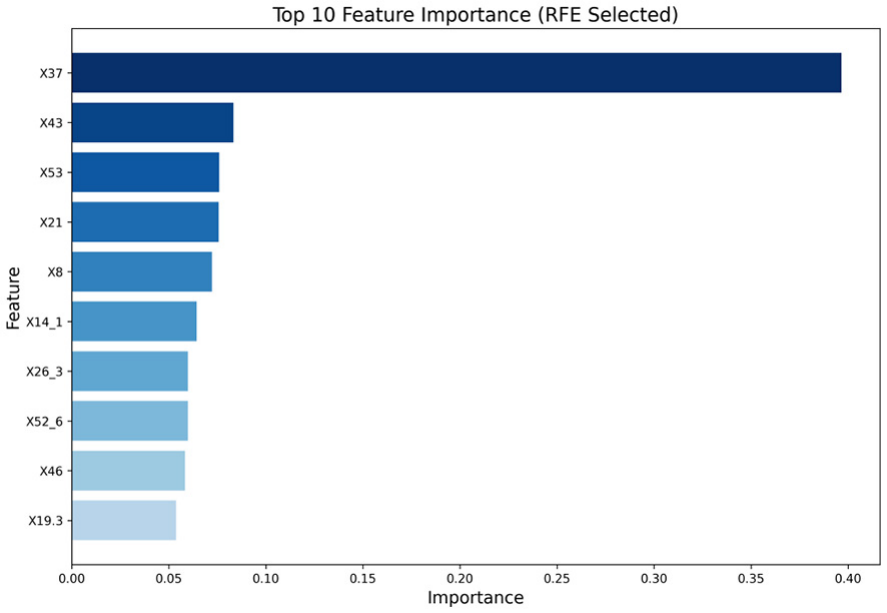
Feature importance ranking after RFECV screening. X37-Did pregnancy weight gain cause distress?; X43-Current maternal sleep condition; X53-Level of social support; X21-Relationship with husband; X8-Work stress during maternity leave; X14_1-Postpartum recovery place-own home; X26_3-Preferred baby gender during pregnancy-one boy, one girl; X52_6-Labor pain relief method-other; X46-Was the mother forced to eat due to breastfeeding?; X19_3-Household members living together-own parents.

After applying RFECV for feature selection on 53 candidate factors, the best feature combination was obtained as 10. The 10-fold cross-validation scores are shown in Figure 2, and the ranking of feature importance is presented in Figure 3.

### Binary logistic regression analysis of early key influencing factors of PPD

We took the result of the postpartum depression screening (whether it was positive or not) as the dependent variable, and the 10 features selected through the random forest feature selection algorithm as the independent variables, and included them in the binary logistic regression analysis. The assignment of independent variables is shown in Table 1. The results of the binary logistic regression analysis indicated that whether there was work pressure during maternity leave, whether forced to eat due to breastfeeding, the relationship between the mother and her husband, whether weight gain during pregnancy was a concern, the current sleep condition of the mother, and the degree of social support was the most crucial influencing factor leading to a positive result in the screening for early postpartum depression (all *P* < 0.05), as shown in Table 2.

**Table 1:** Assignments of independent variables.

Independent variables	Assignment
X8-Work stress during maternity leave	No = 0, Yes = 1
X14_1-Postpartum recovery place-own home	No = 0, Yes = 1
X19_3-Household members living together-own parents	No = 0, Yes = 1
X21-Relationship with husband	Excellent = 1; Good = 2; Average = 3; Poor = 4; Very poor = 5
X26_3-Preferred baby gender during pregnancy-one boy, one girl	No = 0, Yes = 1
X37-Did pregnancy weight gain cause distress?	No = 0, Yes = 1
X43-Current maternal sleep condition	Excellent = 1; Good = 2; Average = 3; Poor = 4; Very poor = 5
X46-Was the mother forced to eat due to breastfeeding?	No = 0, Yes = 1
X52_6-Labor pain relief method-other	No = 0, Yes = 1
X53-Level of social support	High = 1; Average = 2; Low = 3

**Table 2:** Logistic regression analysis of early key influencing factors of postpartum depression.

Variables	β-value	Standard error	*p*-value	OR	95% CI
Constant	−4.570	0.459	< 0.001	0.010	
Work stress during maternity leave	0.503	0.180	0.005	1.654	1.164–2.352
Was the mother forced to eat due to breastfeeding?	0.585	0.212	0.006	1.795	1.185–2.718
Relationship with husband	0.506	0.179	0.005	1.658	1.167–2.356
Did pregnancy weight gain cause distress?	1.940	0.192	< 0.001	6.962	4.778–10.144
Current maternal sleep condition	0.558	0.077	< 0.001	1.748	1.502–2.033
Level of social support	0.404	0.130	0.002	1.498	1.160–1.934

### Performance of each algorithm model and SHAP analysis

**Figure 4:**
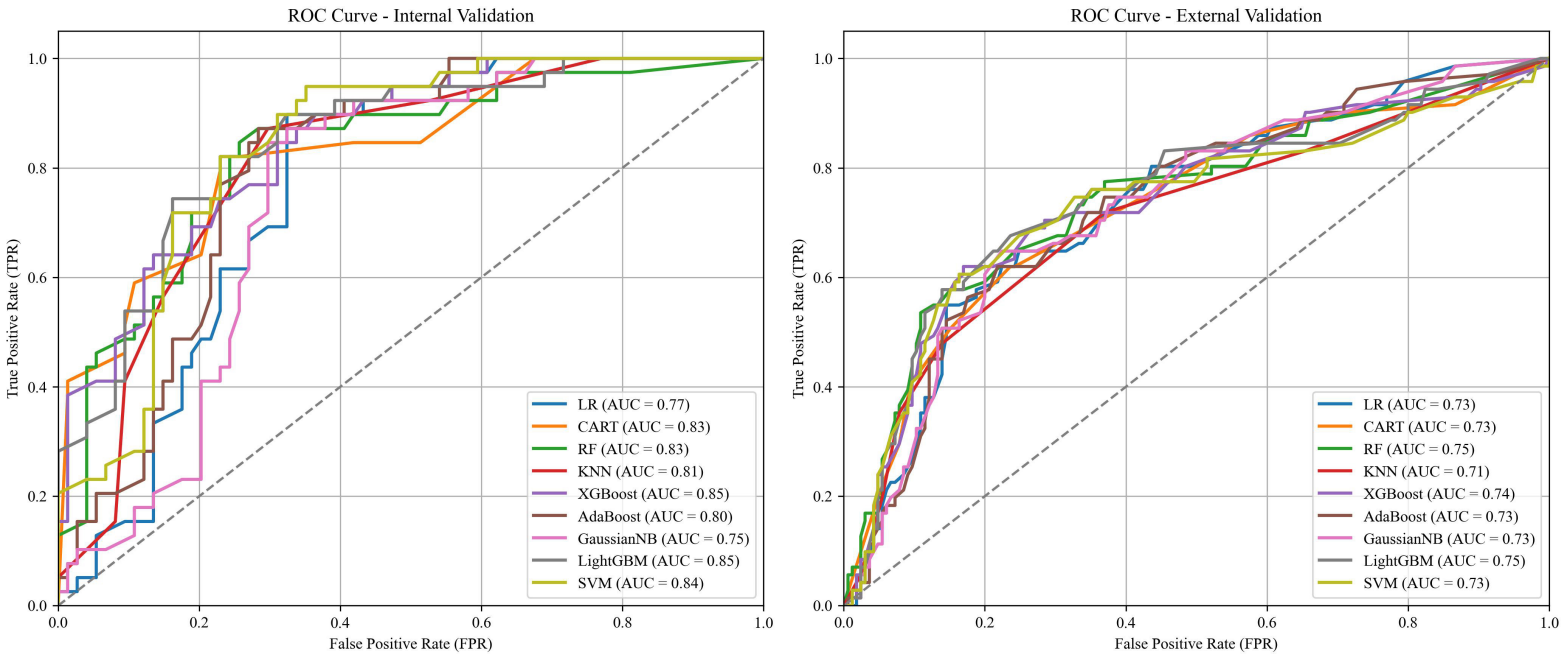
ROC curve comparison between internal and external validation.

**Figure 5:**
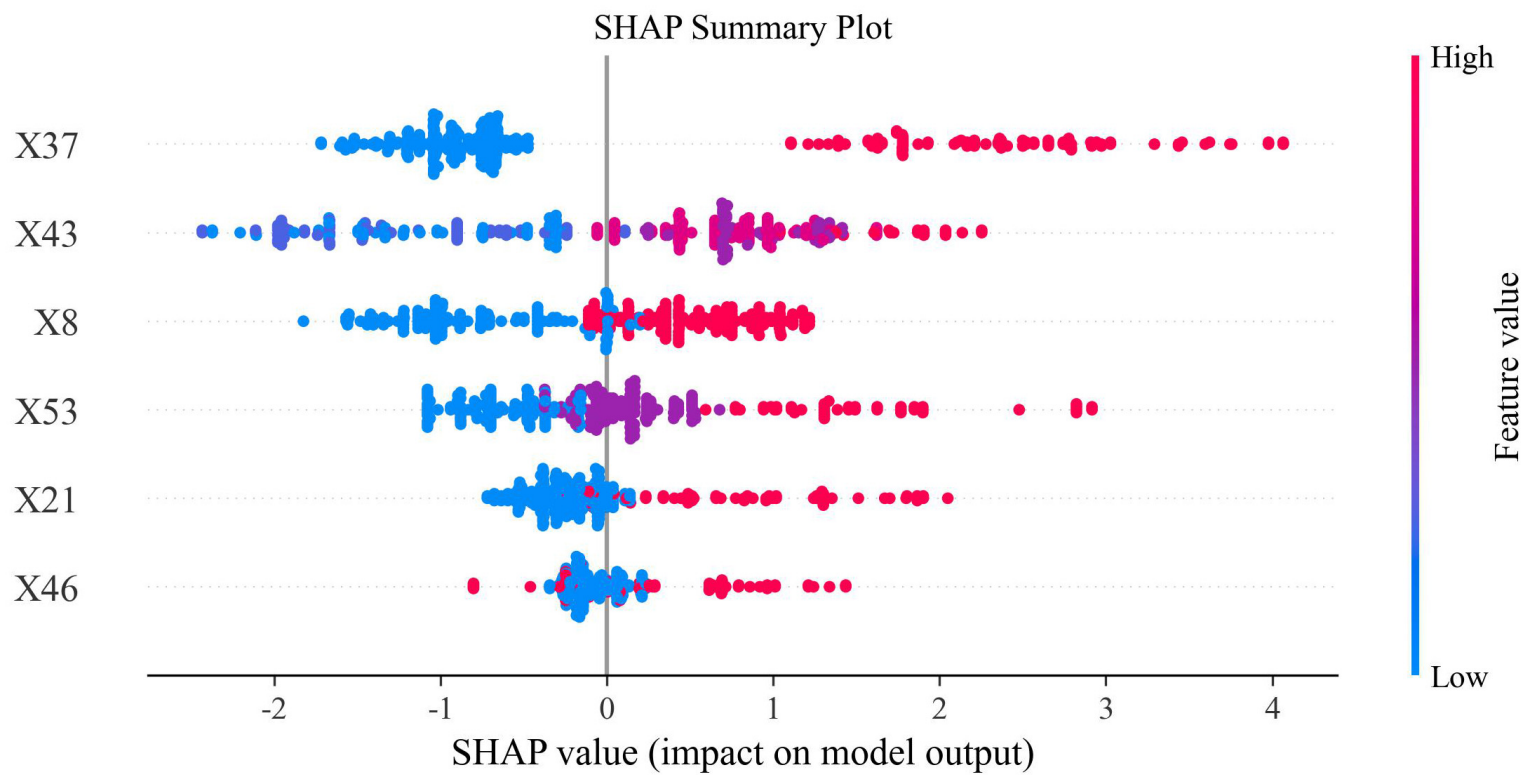
SHAP explainability analysis of the optimal model. X37-Did pregnancy weight gain cause distress?; X43-Current maternal sleep condition, X8-Work stress during maternity leave, X53-Level of social support, X21-Relationship with husband, X46-Was the mother forced to eat due to breastfeeding?

**Figure 6:**
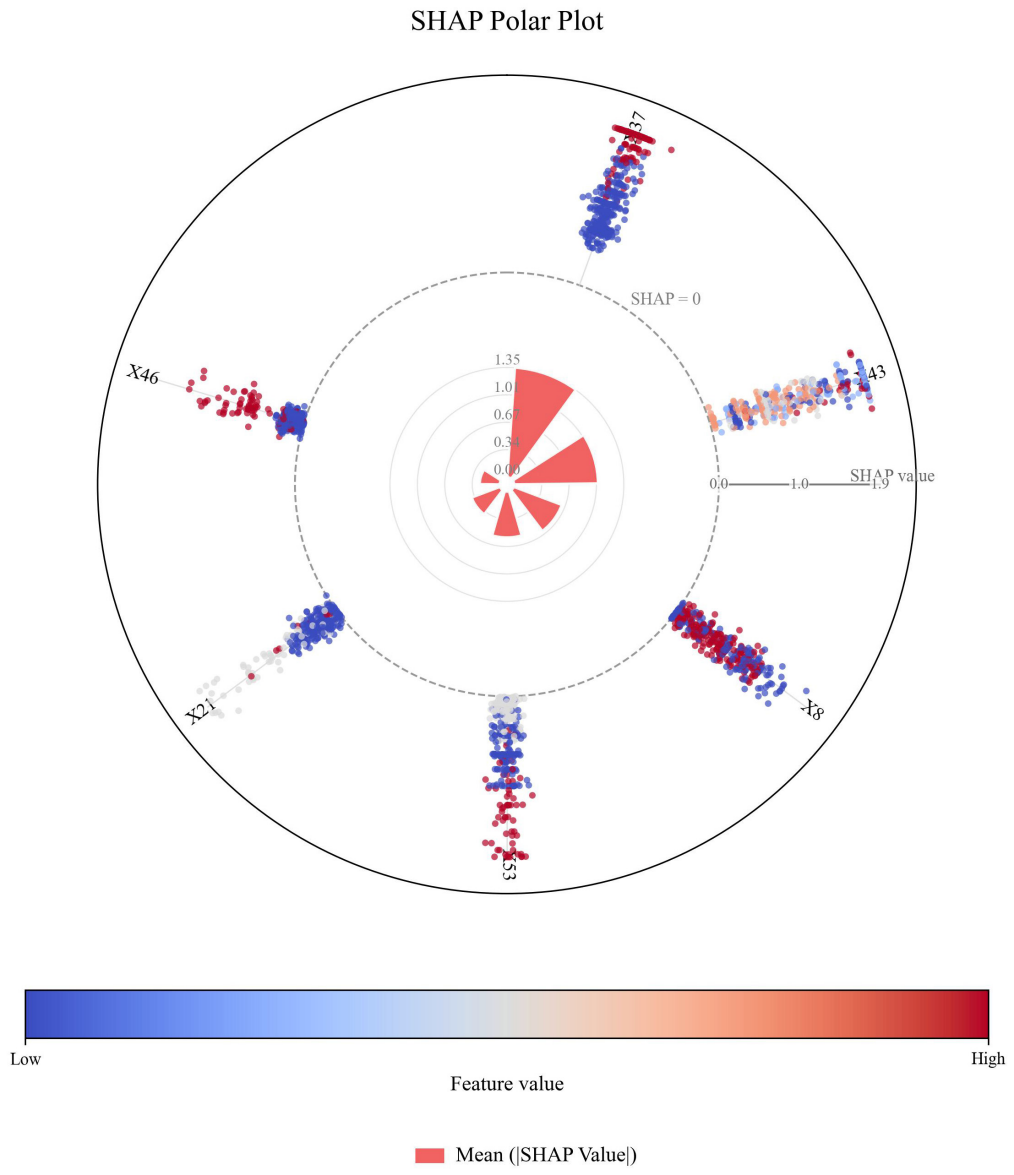
SHAP polar plot of the optimal model. X37-Did pregnancy weight gain cause distress?; X43-Current maternal sleep condition, X8-Work stress during maternity leave, X53-Level of social support, X21-Relationship with husband, X46-Was the mother forced to eat due to breastfeeding?

**Figure 7:**
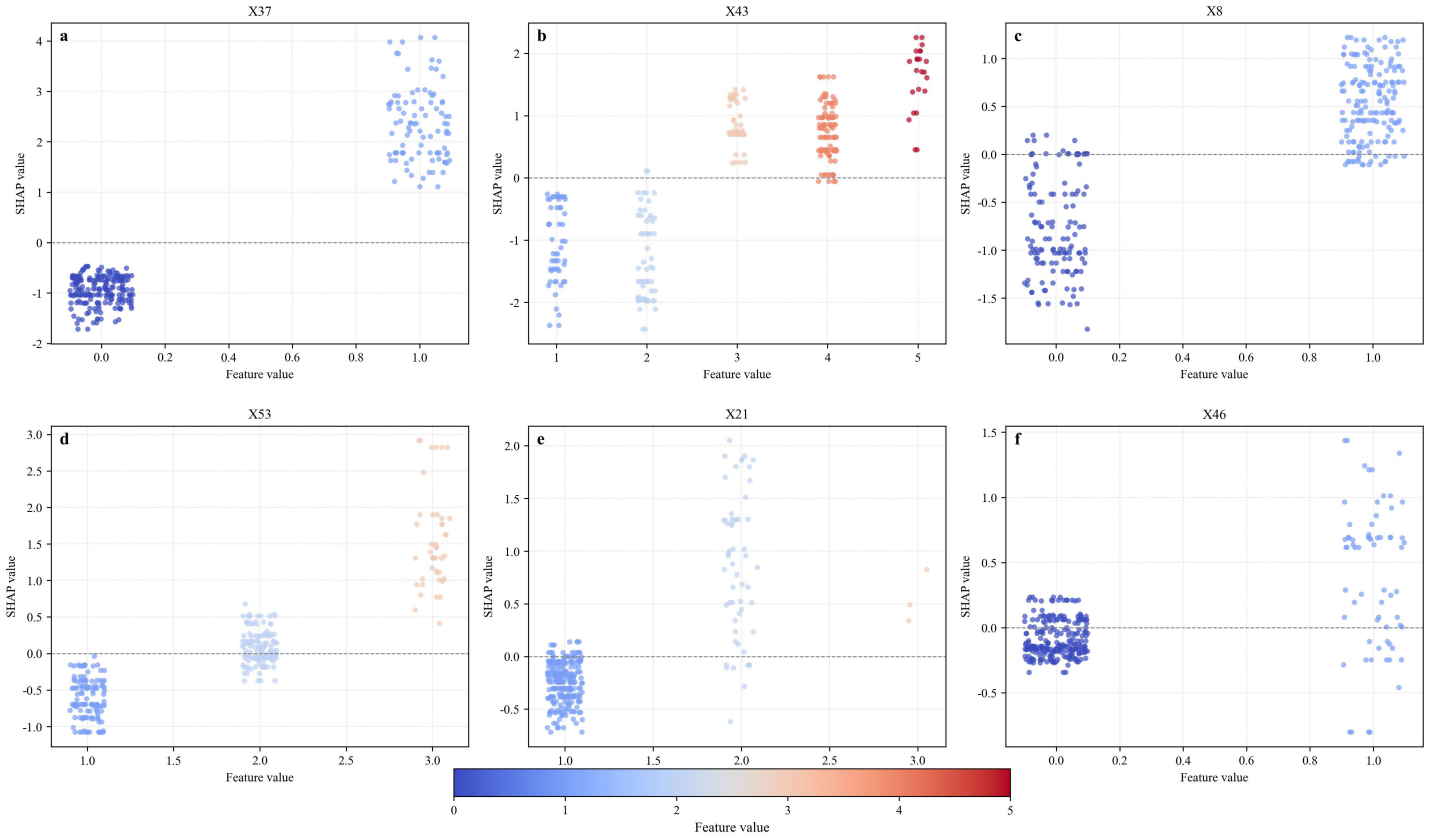
SHAP plot for a single variable of the optimal model. X37-Did pregnancy weight gain cause distress?; X43-Current maternal sleep condition, X8-Work stress during maternity leave, X53-Level of social support, X21-Relationship with husband, X46-Was the mother forced to eat due to breastfeeding?

**Figure 8:**
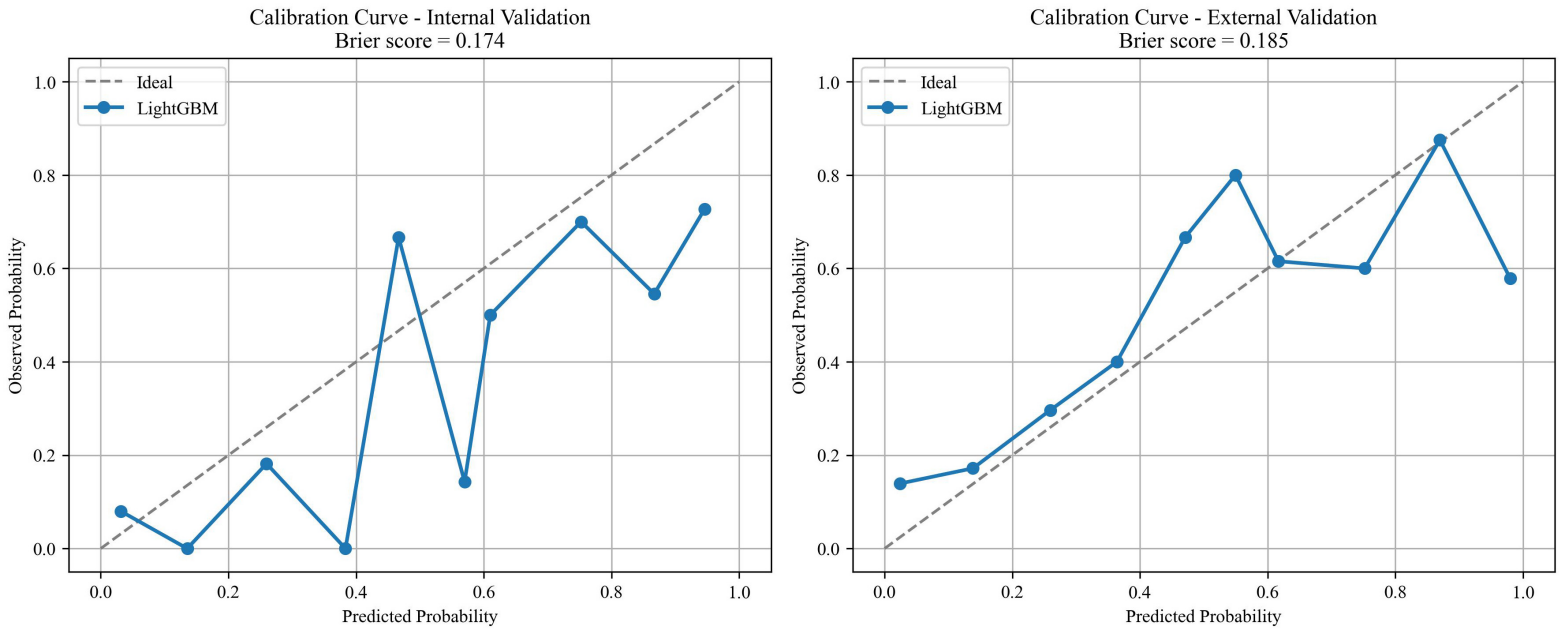
Internal and external validation calibration curve of the optimal model.

**Figure 9:**
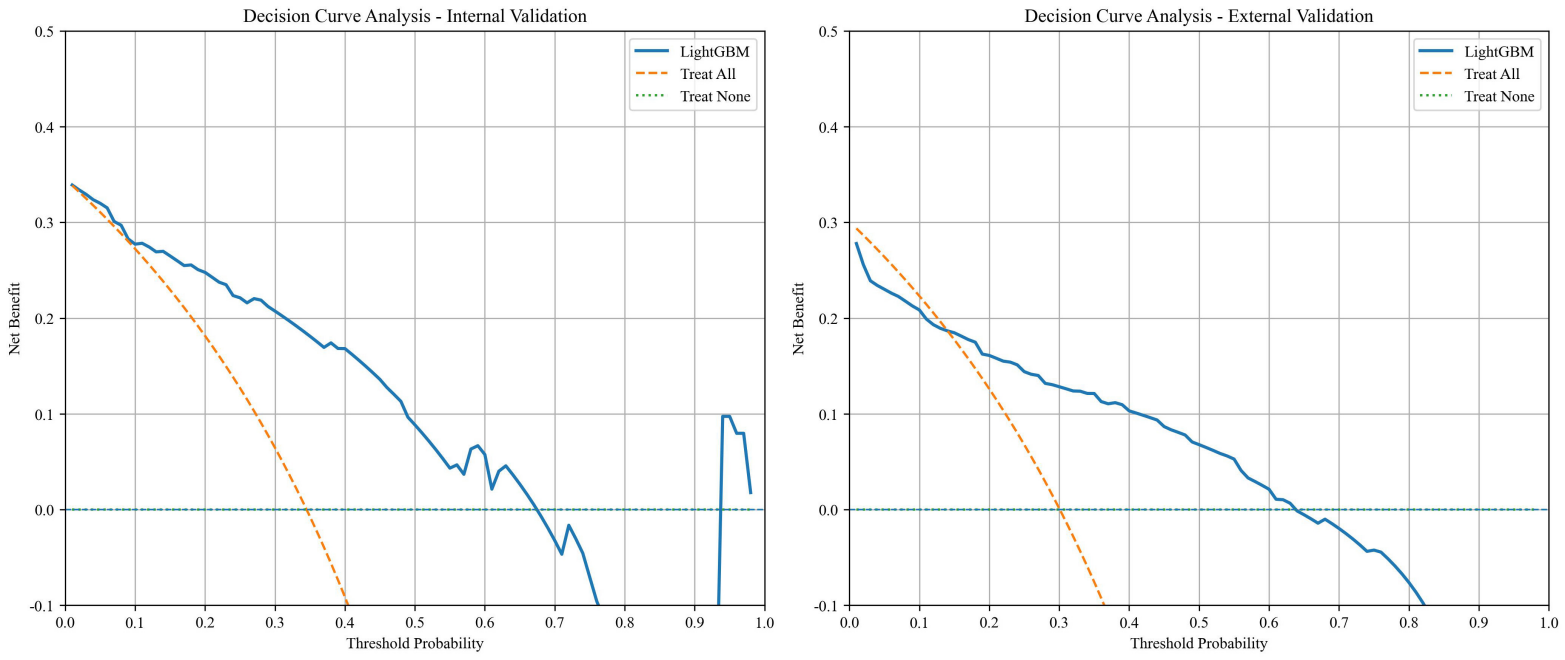
Internal and external validation decision curve analysis of the optimal model.

Six early key influencing factors of PPD were applied to construct nine machine learning algorithm models, and internal and external data were used for verification to obtain various evaluation indicators, as detailed in Tables 3, 4 and Figure 4. By comparison, the optimal algorithm model–LightGBM was obtained and its internal model interpretability was analyzed by SHAP, as shown in Figures 5–7. Figure 8 presents the calibration curves for both internal and external validations, while Figure 9 shows the decision curve analysis for the internal and external validations. The hyperparameters of each model are detailed in the attachment.

**Table 3:** Performance metrics for internal validation across nine algorithms.

Algorithm	Accuracy	Sensitivity	Specificity	F1	AUC	AUC 95% *CI*
Internal validation						
LR	0.752212	0.897436	0.675676	0.714286	0.771656	0.684363–0.849387
CART	0.787611	0.820513	0.770270	0.727273	0.834373	0.747538–0.908887
RF	0.778761	0.846154	0.743243	0.725275	0.830561	0.748158–0.910157
KNN	0.761062	0.871795	0.702703	0.715789	0.814969	0.735464–0.890152
XGBoost	0.743363	0.846154	0.689189	0.694737	0.848926	0.771723–0.911763
AdaBoost	0.769912	0.871795	0.716216	0.723404	0.804574	0.721002–0.883342
GaussianNB	0.752212	0.846154	0.702703	0.702128	0.752945	0.667318–0.840178
LightGBM	0.787611	0.820513	0.770270	0.727273	0.849965	0.771904–0.917566
SVM	0.787611	0.820513	0.770270	0.727273	0.840956	0.767668–0.910465

**Table 4:** Performance metrics for external validation across nine algorithms.

Algorithm	Accuracy	Sensitivity	Specificity	F1	AUC	AUC 95% *CI*
External validation						
LR	0.720339	0.647887	0.751515	0.582278	0.732778	0.663538–0.801342
CART	0.720339	0.619718	0.763636	0.571429	0.729364	0.659871–0.801835
RF	0.766949	0.577465	0.848485	0.598540	0.745540	0.665122–0.814606
KNN	0.656780	0.718310	0.630303	0.557377	0.711140	0.635146–0.783657
XGBoost	0.766949	0.619718	0.830303	0.615385	0.739693	0.663813–0.810480
AdaBoost	0.733051	0.619718	0.781818	0.582781	0.734144	0.661752–0.803758
GaussianNB	0.737288	0.647887	0.775758	0.597403	0.732693	0.663925–0.800562
LightGBM	0.737288	0.676056	0.763636	0.607595	0.746522	0.671237–0.818213
SVM	0.766949	0.605634	0.836364	0.609929	0.732437	0.653636–0.804750

## Discussion

This study, based on the social-ecological system theory, explores the early risk factors for EPDS screening-positive postpartum depressive symptoms from four dimensions: individual, family, community and hospital, and society. By using machine learning methods to analyze the key influencing factors, it ultimately concludes that whether there is work pressure during maternity leave, whether forced to eat due to breastfeeding, the relationship between the mother and her husband, whether weight gain during pregnancy causes distress, the current sleep situation of the mother, and the degree of social support are the early risk factors for EPDS screening-positive postpartum depressive symptoms. The internal and external validations of multiple models confirmed that the LightGBM model performed the best, and the DCA and calibration curves also supported this. A global interpretability analysis of the key influencing factors was conducted using the SHAP algorithm. This study can provide relevant basis for the early precise identification and intervention of postpartum depression in the future.

In this study, the EPDS screening-positive rate in the total sample was 34%, which was consistent with previous research (20). Among them, work pressure during maternity leave was positively correlated with EPDS screening-positive depressive symptoms, indicating that women who encounter work-related stress in the early postpartum period are more likely to develop postpartum depression. This finding is in line with the results of a cohort study (21). For many women, balancing work and family life is often a challenging task, and this pressure can have a negative impact on their mental health (22). An increasing number of studies have shown that excessive work pressure and low job satisfaction are associated with postpartum depression and postpartum anxiety (23, 24), and this association may be mediated by factors such as increased work-family conflict (21). In addition, for older multiparous women, most of whom are career women, they bear heavy work pressure and the pressure of tutoring their children's studies, and sometimes neglect their role as pregnant women, which affects the implementation of health promotion behaviors during pregnancy and leads to the occurrence of postpartum depression (25). At this time, the sharing from family members, especially the husband, often increases the happiness of the parturient and reduces her psychological pressure (26), thereby, to some extent, alleviating the occurrence or development of postpartum depression.

The dietary patterns of postpartum women often undergo significant changes due to the traditional custom of “sitting in confinement” (27). In this study, it was found that postpartum women who were forced to eat due to breastfeeding were positively correlated with the EPDS screening-positive depressive symptoms. During this period, mothers are often forced to consume large amounts of nutrients to ensure breastfeeding and body recovery after childbirth, and are required to reduce the intake of cold foods to maintain the balance of qi and blood in the body (28). In addition, studies have shown (29) that a dietary pattern of consuming large amounts of meat and eggs while reducing the intake of vegetables, mushrooms, and nuts is most similar to the postpartum dietary pattern in China, and this pattern is more likely to cause postpartum depression. Such dietary changes also pose a risk of inflammation to the body (30), exacerbating the occurrence of negative emotions. The results of this study also show that the relationship between postpartum women and their husbands, that is, the marital relationship, is positively correlated with EPDS screening-positive depressive symptoms. This result is supported by many studies (31–33), and some studies have shown that a poor marital relationship is a major influencing factor for postpartum depression (32). This factor may be affected by issues such as postpartum hormone level fluctuations, parenting pressure, economic conditions, and role anxiety. At this stage, the psychology of postpartum women is more fragile and more susceptible to the influence of their partners. If the marital relationship is poor at this time, postpartum women often experience a greater sense of hopelessness and helplessness (31), thereby exacerbating the occurrence of postpartum depression. In addition, some studies have also found (34) that postpartum depression can further deteriorate the marital relationship, indicating that the two may be influencing factors for each other.

This study also revealed a positive correlation between whether weight gain during pregnancy causes distress and EPDS screening-positive depressive symptoms. During the investigation, some pregnant women did not experience anxiety or stress due to weight changes (whether increase or decrease), while others were more concerned about weight fluctuations despite no significant changes in their weight. This phenomenon is also supported by relevant research (35, 36), indicating that this perception of weight-related stress may be a contributing factor to postpartum depression. Additionally, this study confirmed that current sleep conditions of mothers were associated with postpartum depression, with poorer status corresponding to higher risk. A previous meta-analysis (37) has similarly demonstrated that longer sleep duration in the postpartum period is associated with reduced fatigue and fewer depressive symptoms. In addition, other studies (38) have reported a strong association between subjective sleep quality and the severity of depressive symptoms. Insufficient sleep after childbirth has also been linked to an increased risk of postpartum depressive symptoms, as well as adverse health outcomes such as coronary heart disease, diabetes, and obesity (39–42). Finally, the results of this study also confirmed a positive correlation between the degree of social support and EPDS screening-positive depressive symptoms, a finding that is fully supported by other research (43). Previous studies have further examined the role of social support (44), demonstrating that partner support is a key predictor of postpartum depressive symptoms, while support from friends is also associated with symptom severity. In addition, insufficient partner support may substantially increase the burden of childcare and further aggravate postpartum depressive symptoms (45).

Ultimately, we conducted SHAP analysis based on the best model (namely the LightGBM algorithm model), and visualized the results in the form of multiple swarm plots and a Polar Plot (Figures 5–7). This plot shows the global feature importance ranking within the model, the influence direction and distribution of each key influencing factor, as well as the interaction pattern between feature values and influences. The vertical axis of the beeswarm plot lists all the features input into the model, and the features are arranged in descending order of importance. This ranking is based on the average absolute SHAP value. The horizontal axis represents the SHAP value itself, which directly reflects the contribution direction and intensity of each feature to individual prediction results (46). In the plot, the color of each point typically represents the original value size of the feature (for example, red represents high values and blue represents low values).

Figures 5 extbfand 6 illustrate the global interpretability of the optimal prediction model. Among all predictors, distress related to gestational weight gain demonstrated the largest contribution to model-predicted risk, as reflected by its top importance ranking and the widest SHAP value distribution. The feature exhibited substantial dispersion across both negative and positive SHAP values, with an extended positive tail, indicating marked heterogeneity in its contribution to model predictions across individuals. This pattern suggests that the modeled relationship between weight-related distress and predicted risk may be non-linear and potentially interactive with other psychosocial variables, resulting in differential contribution patterns across subgroups.

In contrast, sleep quality and marital relationship were predominantly distributed on the negative side of the SHAP axis when feature values were favorable (blue points), indicating substantial negative contributions to predicted risk within the model. A gradient pattern was observed whereby poorer sleep status and marital discord (red points) were associated with increasing positive SHAP values. Social support demonstrated a similar, though comparatively smaller, contribution pattern. Although work pressure during maternity leave and breastfeeding-related forced eating ranked lower in overall importance, their SHAP distributions indicate that they remain relevant contributors to model-predicted risk. The distribution of a single factor is shown in Figure 7. Collectively, these findings highlight six key early psychosocial predictors identified by the model that may inform risk stratification, early screening prioritization, and targeted intervention planning within hospital-based perinatal mental health management pathways.

Despite the promising predictive performance and interpretability of the model, several challenges may arise during real-world clinical implementation. Integrating machine learning–assisted screening into routine obstetric workflows requires alignment with existing clinical processes, including data collection timing, staff responsibilities, and electronic medical record interoperability. In busy maternity wards, additional screening tasks may increase the workload of nurses and obstetric staff, particularly in settings with limited workforce capacity or mental health training. Furthermore, the feasibility of real-time risk calculation depends on the availability and standardization of psychosocial data, some of which are not routinely captured in structured hospital records. To enhance practical applicability, future implementation efforts should focus on embedding automated risk calculators within hospital information systems, streamlining questionnaire administration, and establishing clear referral pathways for high-risk mothers. Addressing these workflow and feasibility considerations will be essential for translating model outputs into sustainable, routine perinatal mental health screening practice.

## Limitations

First, participants were recruited from two tertiary grade-A general hospitals in Shandong Province, China; therefore, findings may not be directly generalizable to primary care facilities, rural hospitals, or regions with different healthcare resources and service delivery systems. Tertiary hospitals in China typically differ from lower-tier institutions in organizational structure, staffing capacity, perinatal mental health resources, and standardized screening workflows. These structural and operational differences may influence both the feasibility of implementing machine learning-assisted screening pathways and the distribution or relative importance of psychosocial risk factors across settings.

Second, several predictors were self-reported psychosocial variables, which may be subject to recall bias and social desirability bias. Future studies should expand to multi-center, larger, and more diverse samples, extend follow-up beyond 4–6 weeks postpartum to evaluate longer-term trajectories, and consider incorporating objective measures (e.g., actigraphy-based sleep metrics or biological markers) to enhance measurement robustness.

Finally, the self-developed questionnaire in this study was used to assist in collecting potential relevant influencing factors for this research. Therefore, no psychometric validation was conducted. Additionally, in the feature selection process, the internal validation subset was not completely independent during the feature selection stage, which may pose a risk of information leakage.

## Conclusion

In conclusion, this study, based on the social-ecological system theory, explores the early influencing factors of postpartum depression from multiple dimensions and perspectives. It uses machine learning methods to accurately identify the key early influencing factors and compares to obtain the best machine learning algorithm model. Combined with SHAP analysis for global interpretability of the model's internal structure, it can provide reliable references for the future realization of early precise prediction and intervention of postpartum depression in hospitals.

## Author's note

The data used in this study is consistent with that of previous published studies. For details, please refer to doi: 10.3389/fpubh.2025.1685305.

All authors confirm that the following manuscript is a transparent and honest account of the reported research. This research is related to a previous study by the same authors titled, “Development and validation of an interpretable machine learning model and online web-based calculator based on social-ecosystem theory for early prediction of postpartum depression: a longitudinal study”. The previous study was conducted using the same dataset and guided by the same theoretical framework.

However, the current submission differs substantially in several important aspects. First, regarding the research objective, the previous study primarily focused on constructing an early prediction model, followed by risk stratification and the development of an online calculator, with an emphasis on practical web-based application. In contrast, the present study focuses on a methodological re-analysis within the same framework, highlighting RFECV-based feature selection and interpretable modeling, with the aim of identifying early key influencing factors while reducing subjective selection bias.

Second, in terms of feature selection, the previous study adopted a combination of univariate analysis and binary logistic regression, whereas the current study employs one-hot encoding combined with RFECV and logistic regression. This approach provides a more data-driven and objective variable selection process that is more consistent with machine learning modeling principles. Third, with respect to model evaluation, the present study incorporates additional evaluation methods, including decision curve analysis (DCA) and calibration curves, to assess predictive accuracy, clinical utility, and net benefit.

Fourth, regarding interpretability, the current study extends SHAP analysis by including single-feature dependence plots and SHAP Polar Plots, allowing for more comprehensive visualization of both global and local model explanations. Finally, the predictors identified in the two studies differ. The previous study reported seven relevant factors, whereas the current study identified six. Notably, the factor “forced eating due to breastfeeding” is of particular significance in the present study and has not been directly identified in previous research.

The study is following the methodology explained in the previous work while incorporating methodological refinements and additional analyses. Both studies provide complementary insights and may contribute to the future development of early risk management strategies for postpartum depressive symptoms.

## Data Availability

The raw data supporting the conclusions of this article will be made available by the authors, without undue reservation.
