# Supplementary material for: Identifying early key influencing factors of positive results in the early screening for postpartum depression with interpretable machine learning
Source: Front Public Health. 2026 May 15;14:1761983. doi: 10.3389/fpubh.2026.1761983 (PMC13228117; doi:10.3389/fpubh.2026.1761983)
Supplement: Supplementary file 1 [file Data_Sheet_1.docx]

***Appendice***

**Table S1** Descriptive analysis of the early influencing factors of the training set and validation set.

**Table S2** Assignments of independent variables.

**Table S3** Diagnosis of collinearity.

**Table S4** All models hyperparameters

**Table S1** Descriptive analysis of the early influencing factors of the training set and validation set.

| **Variables** | **Training set**  **(n=563)** | **Validation set**  **(n=236)** |
| --- | --- | --- |
| X1-Maternal age,years | 32.02±4.86 | 31.68±4.17 |
| X2-Husband’s age,years | 34.11±6.71 | 32.92±4.35 |
| X3-Maternal education |  |  |
| Primary school and below  Junior high school or secondary school  High school or college  Undergraduate  Master’s degree or above | 0(0.0)  47(8.3)  196(34.8)  211(37.5)  109(19.4) | 0(0.0)  18(7.6)  55(23.3)  108(45.8)  55(23.3) |
| X4-Husband’s education |  |  |
| Primary school and below  Junior high school or secondary school  High school or college  Undergraduate  Master's degree or above | 2(0.4)  51(9.1)  159(28.2)  251(44.6)  100(17.8) | 0(0.0)  17(7.2)  60(25.4)  113(47.9)  46(19.5) |
| X5-Marital status |  |  |
| Unmarried  First marriage  Remarriage  Get divorced  Widowed spouse | 7(1.2)  537(95.4)  19(3.4)  0(0.0)  0(0.0) | 3(1.3)  227(96.2)  6(2.5)  0(0.0)  0(0.0) |
| X6-Maternal Pre-Pregnancy Average Monthly Income |  |  |
| No income  < 3,000 CNY  3,000-5,999 CNY  6,000-9,999 CNY  10,000-20,000 CNY  > 20,000 CNY | 49(8.7)  19(3.4)  168(29.8)  242(43.0)  72(12.8)  13(2.3) | 11(4.7)  10(4.2)  68(28.8)  108(45.8)  34(14.4)  5(2.1) |
| X7-Maternity Leave Duration |  |  |
| < 1 month  1-3 months  4-6 months  7-8 months  9-12 months  More than 1 year | 18(3.2)  44(7.8)  410(72.8)  14(2.5)  2(0.4)  75(13.3) | 12(5.1)  13(5.5)  176(74.6)  9(3.8)  1(0.4)  25(10.6) |
| X8-Work Stress During Maternity Leave |  |  |
| No  Yes | 250(44.4)  313(55.6) | 194(82.2)  42(17.8) |
| X9-Maternity Insurance |  |  |
| No  Yes | 83(14.7)  480(85.3) | 28(11.9)  208(88.1) |
| X10-Stable Income During Maternity Leave |  |  |
| No  Yes | 148(26.3)  415(73.7) | 57(24.2)  179(75.8) |
| X11-Husband’s Average Monthly Income |  |  |
| No income  < 3,000 CNY  3,000-5,999 CNY  6,000-9,999 CNY  10,000-20,000 CNY  ＞20,000 CNY | 2(0.4)  7(1.2)  103(18.3)  221(39.3)  173(30.7)  57(10.1) | 2(0.8)  6(2.5)  45(19.1)  88(37.3)  72(30.5)  23(9.7) |
| X12-Husband’s Care Leave |  |  |
| < 1 week  1-2 weeks  3-4 weeks  4-8 weeks  More than 8 weeks | 100(17.8)  321(57.0)  66(11.7)  18(3.2)  58(10.3) | 39(16.5)  130(55.1)  35(14.8)  7(3.0)  25(10.6) |
| X13-Can the Husband Provide Sufficient Care? |  |  |
| Yes  No | 483(85.8)  80(14.2) | 213(90.3)  23(9.7) |
| X14-Postpartum Recovery Place |  |  |
| Own home  Postpartum care center  Others | 476(84.5)  44(7.8)  43(7.6) | 104(44.1)  114(48.3)  18(7.6) |
| X15-Maternal Evaluation of Living Place |  |  |
| Spacious and comfortable  Average  Poor conditions  Very poor conditions | 440(78.2)  121(21.5)  2(0.4)  0(0.0) | 198(83.9)  38(16.1)  0(0.0)  0(0.0) |
| X16-Are Community Neighborly Relations Harmonious? |  |  |
| No  Yes | 8(1.4)  555(98.6) | 1(0.4)  235(99.6) |
| X17-Is Medical Care Convenient at the Place of Residence? |  |  |
| No  Yes | 58(10.3)  505(89.7) | 18(7.6)  218(92.4) |
| X18-Maternal Evaluation of Designated Maternity Healthcare Facility |  |  |
| Excellent  Good  Average  Poor  Very poor | 427(75.8)  105(18.7)  31(5.5)  0(0.0)  0(0.0) | 179(75.8)  42(17.8)  14(5.9)  1(0.4)  0(0.0) |
| X19-Household Members Living Together  X19.1 Husband and Children |  |  |
| No  Yes | 97(17.2)  466(82.8) | 24(10.2)  212(89.8) |
| X19.2 Husband’s Parents |  |  |
| No  Yes | 384(68.2)  179(31.8) | 163(69.1)  73(30.9) |
| X19.3 Own Parents |  |  |
| No  Yes | 463(82.2)  100(17.8) | 184(78.0)  52(22.0) |
| X19.4 Husband’s Siblings |  |  |
| No  Yes | 559(99.3)  4(0.7) | 236(100.0)  0(0.0) |
| X19.5 Wife’s Siblings |  |  |
| No  Yes | 561(99.6)  2(0.4) | 234(99.2)  2(0.8) |
| X19.6 Maternity Matron or Nanny |  |  |
| No  Yes | 506(89.9)  57(10.1) | 188(79.7)  48(20.3) |
| X19.7 Others |  |  |
| No  Yes | 548(97.3)  15(2.7) | 233(98.7)  3(1.3) |
| X20-Maternal Evaluation of Family Relationships |  |  |
| Excellent  Good  Average  Poor  Very poor | 386(68.6)  142(25.2)  27(4.8)  8(1.4)  0(0.0) | 185(78.4)  46(19.5)  5(2.1)  0(0.0)  0(0.0) |
| X21-Relationship with Husband |  |  |
| Excellent  Good  Average  Poor  Very poor | 442(78.5)  112(19.9)  0(0.0)  0(0.0)  9(1.6) | 196(83.1)  37(15.7)  0(0.0)  0(0.0)  3(1.3) |
| X22-Relationship with Parents-in-law |  |  |
| Excellent  Good  Average  Poor  Very poor | 349(62.0)  159(28.2)  54(9.6)  1(0.2)  0(0.0) | 160(67.8)  59(25.0)  14(5.9)  3(1.3)  0(0.0) |
| X23-Relationship with Parents |  |  |
| Excellent  Good  Average  Poor  Very poor | 412(73.2)  131(23.3)  14(2.5)  0(0.0)  6(1.1) | 191(80.9)  41(17.4)  3(1.3)  0(0.0)  1(0.4) |
| X24-Was This Pregnancy Planned? |  |  |
| No  Yes | 56(9.9)  507(90.1) | 25(10.6)  211(89.4) |
| X25-Parity |  |  |
| First birth  Second birth  Third birth  More than three births | 408(72.5)  138(24.5)  15(2.7)  2(0.4) | 189(80.1)  45(19.1)  2(0.8)  0(0.0) |
| X26-Preferred Baby Gender During Pregnancy |  |  |
| Boy  Girl  One boy, one girl  Two girls  Two boys  Multiple births  No preference | 72(12.8)  109(19.4)  38(6.7)  0(0.0)  0(0.0)  2(0.4)  342(60.7) | 62(26.3)  85(36.0)  34(14.4)  0(0.0)  0(0.0)  2(0.8)  53(22.5) |
| X27-Maternal Attitude Toward Actual Baby Gender |  |  |
| Very satisfied  No preference  Not satisfied | 399(70.9)  5(0.9)  159(28.2) | 169(71.6)  37(15.7)  30(12.7) |
| X28-Do Family Members Have Gender Bias Toward the Baby? |  |  |
| No  Yes | 9(1.6)  554(98.4) | 137(58.1)  99(41.9) |
| X29-Has the Mother Experienced Domestic Violence? |  |  |
| No  Yes | 556(98.8)  7(1.2) | 234(99.2)  2(0.8) |
| X30-Can the Family Provide Sufficient Daily Life and Care Support? |  |  |
| No  Yes | 11(2.0)  552(98.0) | 5(2.1)  231(97.9) |
| X31-Can the Family Provide Sufficient Infant Care Support? |  |  |
| No  Yes | 23(4.1)  540(95.9) | 6(2.5)  230(97.5) |
| X32-Does the Mother Have Someone to Confide in When Conflicts Arise with Family Members? |  |  |
| No  Yes | 59(10.5)  504(89.5) | 11(4.7)  225(95.3) |
| X33-Was Folic Acid Supplemented Regularly During Pregnancy? |  |  |
| No  Regularly  Irregularly | 254(45.1)  242(43.0)  67(11.9) | 96(40.7)  125(53.0)  15(6.4) |
| X34-Regular prenatal care |  |  |
| No  Yes | 11(2.0)  552(98.0) | 0(0.0)  236(100.0) |
| X35-Pre-Pregnancy BMI | 24.07±6.40 | 23.4±5.70 |
| X36-Pre-Delivery BMI | 29.48±7.50 | 28.5±6.6 |
| X37-Did Pregnancy Weight Gain Cause Distress? |  |  |
| No  Yes | 389(69.1)  174(30.9) | 173(73.3)  63(26.7) |
| X38-Whether the following conditions occurred during pregnancy |  |  |
| X38.1 Gestational Diabetes |  |  |
| No  Yes | 441(78.3)  122(21.7) | 198(83.9)  38(16.1) |
| X38.2 Gestational Hypertension |  |  |
| No  Yes | 536(95.2)  27(4.8) | 219(92.8)  17(7.2) |
| X38.3 Hyperthyroidism |  |  |
| No  Yes | 553(98.2)  10(1.8) | 231(97.9)  5(2.1) |
| X38.4 Hypothyroidism |  |  |
| No  Yes | 525(93.3)  38(6.7) | 224(94.9)  12(5.1) |
| X38.5 Threatened Miscarriage |  |  |
| No  Yes | 507(90.1)  56(9.9) | 211(89.4)  25(10.6) |
| X38.6 Threatened Preterm Labor |  |  |
| No  Yes | 522(92.7)  41(7.3) | 222(94.1)  14(5.9) |
| X38.7 Other Conditions |  |  |
| No  Yes | 530(94.1)  33(5.9) | 224(94.9)  12(5.1) |
| X39-Low birth weight |  |  |
| No  Yes | 524(93.1)  39(6.9) | 224(94.9)  12(5.1) |
| X40-Is the Newborn Healthy? |  |  |
| No  Yes | 12(2.1)  551(97.9) | 2(0.8)  234(99.2) |
| X41-Current Baby Feeding Method |  |  |
| Breastfeeding  Mixed feeding  Formula feeding | 208(36.9)  301(53.5)  54(9.6) | 70(29.7)  145(61.4)  21(8.9) |
| X42-Baby’s Characteristics |  |  |
| Well-behaved  Easily cries  Difficult to feed | 442(78.5)  109(19.4)  12(2.1) | 185(78.4)  42(17.8)  9(3.8) |
| X43-Current Maternal Sleep Condition |  |  |
| Excellent  Good  Average  Poor  Very poor | 106(18.8)  126(22.4)  99(17.6)  187(33.2)  0(0.0) | 53(22.5)  51(21.6)  45(19.1)  75(31.8)  12(5.1) |
| X44-Maternal Confidence in Newborn Care |  |  |
| Very confident  Fairly confident  Average confidence  No confidence | 278(49.4)  218(38.7)  58(10.3)  9(1.6) | 122(51.7)  95(40.3)  18(7.6)  1(0.4) |
| X45-Does Postpartum Diet Meet Personal Preferences? |  |  |
| No  Yes | 74(13.1)  489(86.9) | 27(11.4)  209(88.6) |
| X46-Was the Mother Forced to Eat Due to Breastfeeding? |  |  |
| No  Yes | 443(78.7)  120(21.3) | 197(83.5)  39(16.5) |
| X47-Personality Type  X47.1 Extroverted |  |  |
| No  Yes | 353(62.7)  210(37.3) | 147(62.3)  89(37.7) |
| X47.2 Introverted |  |  |
| No  Yes | 457(81.2)  106(18.8) | 168(71.2)  68(28.8) |
| X47.3 Mixed |  |  |
| No  Yes | 342(60.7)  221(39.3) | 177(75.0)  59(25.0) |
| X47.4 Stable |  |  |
| No  Yes | 344(61.1)  219(38.9) | 159(67.4)  77(32.6) |
| X47.5 Unstable |  |  |
| No  Yes | 512(90.9)  51(9.1) | 191(80.9)  45(19.1) |
| X48-Time from Regular Contractions to Delivery |  |  |
| < 4 hours  4-6 hours  > 6 hours | 267(47.4)  166(29.5)  130(23.1) | 140(59.3)  47(19.9)  49(20.8) |
| X49-Feelings During Delivery  X49.1 Excited |  |  |
| No  Yes | 483(85.8)  80(14.2) | 145(61.4)  91(38.6) |
| X49.2 Nervous |  |  |
| No  Yes | 234(41.6)  329(58.4) | 165(69.9)  71(30.1) |
| X49.3 Painful |  |  |
| No  Yes | 250(44.4)  313(55.6) | 97(41.1)  139(58.9) |
| X49.4 Happy |  |  |
| No  Yes | 447(79.4)  116(20.6) | 148(62.7)  88(37.3) |
| X49.5 Other |  |  |
| No  Yes | 537(95.4)  26(4.6) | 194(82.2)  42(17.8) |
| X50-Family history of mental illness |  |  |
| No  Yes | 535(95.0)  28(5.0) | 231(97.9)  5(2.1) |
| X51-Mode of delivery |  |  |
| Vaginal delivery  Cesarean delivery  Painless delivery | 186(33.0)  365(64.8)  12(2.1) | 77(32.6)  148(62.7)  11(4.7) |
| X52-Labor Pain Relief Method |  |  |
| None  Epidural analgesia  Intravenous analgesia  Non-pharmacological pain relief  General anesthesia  Other | 99(17.6)  376(66.8)  15(2.7)  0(0.0)  55(9.8)  18(3.2) | 43(18.2)  146(61.9)  14(5.9)  0(0.0)  22(9.3)  11(4.7) |
| X53-Level of Social Support |  |  |
| High  Average  Low | 228(40.5)  254(45.1)  81(14.4) | 113(47.9)  106(44.9)  17(7.2) |
| EPDS Score  ≥ 10  < 10 | 362(64.3)  201(35.7) | 165(70.0)  71(30.0) |

**Table S2** Assignments of independent variables.

| **Independent variables** | **Assignment** |
| --- | --- |
| X8-Work Stress During Maternity Leave | No=0,Yes=1 |
| X13-Can the Husband Provide Sufficient Care? | Yes=1,No=2 |
| X14-Postpartum Recovery Place | Own home(Z1=0,Z2=0);Postpartum care center(Z1=1,Z2=0);Others(Z1=0,Z2=1) |
| X15-Maternal Evaluation of Living Place | Spacious and comfortable=1;Average=2;Poor conditions=3;Very poor conditions=4 |
| X20-Maternal Evaluation of Family Relationships | Excellent=1;Good=2;Average=3;Poor=4;Very poor=5 |
| X21-Relationship with Husband | Excellent=1;Good=2;Average=3;Poor=4;Very poor=5 |
| X22-Relationship with Parents-in-law | Excellent=1;Good=2;Average=3;Poor=4;Very poor=5 |
| X23-Relationship with Parents | Excellent=1;Good=2;Average=3;Poor=4;Very poor=5 |
| X37-Did Pregnancy Weight Gain Cause Distress? | No=0,Yes=1 |
| X38.6-Whether the following conditions occurred during pregnancy-Threatened Preterm Labor | No=0,Yes=1 |
| X43-Current Maternal Sleep Condition | Excellent=1;Good=2;Average=3;Poor=4;Very poor=5 |
| X46-Was the Mother Forced to Eat Due to Breastfeeding? | No=0,Yes=1 |
| X47.2-Personality Type-Introverted | No=0,Yes=1 |
| X49.1-Feelings During Delivery-Excited | No=0,Yes=1 |
| X53-Level of Social Support | High=1;Average=2;Low=3 |

**Table S3** Diagnosis of collinearity.

| **Independent variables** | **VIF** |
| --- | --- |
| X8-Work Stress During Maternity Leave | 1.031 |
| X13-Can the Husband Provide Sufficient Care? | 1.206 |
| X14-Postpartum Recovery Place | 1.002 |
| X22-Relationship with Parents-in-law | 1.005 |
| X37-Did Pregnancy Weight Gain Cause Distress? | 1.005 |
| X43-Current Maternal Sleep Condition | 1.009 |
| X53-Level of Social Support | 1.226 |

**Table S4** All models hyperparameters.

| Models | hyperparameters |
| --- | --- |
| LR | C = 10 |
| CART | max_depth = 5 |
| RF | max_depth = 10, n_estimators = 200 |
| KNN | n_neighbors = 7 |
| XGBoost | learning_rate = 0.05, max_depth = 5 |
| AdaBoost | n_estimators = 100 |
| GaussianNB | No hyperparameters tuned |
| LightGBM | learning_rate = 0.1, n_estimators = 200, num_leaves = 31 |
| SVM | C = 10 |
